# An Updated Systematic Review and Meta-Analysis of the Obstetric Consequences of Female Genital Mutilation/Cutting

**DOI:** 10.1155/2014/542859

**Published:** 2014-11-23

**Authors:** R. C. Berg, J. Odgaard-Jensen, A. Fretheim, V. Underland, G. Vist

**Affiliations:** The Norwegian Knowledge Centre for the Health Services, P.O. Box 7004, St.Olavs Plass, 0130 Oslo, Norway

## Abstract

In our recent systematic review in *Obstetrics and Gynecology International* of the association between FGM/C and obstetric harm we concluded that FGM/C significantly increases the risk of delivery complications. The findings were based on unadjusted effect estimates from both prospective and retrospective studies. To accommodate requests by critics, we aimed to validate these results through additional analyses based on adjusted estimates from prospective studies. We judged that 7 of the 28 studies included in our original systematic review were prospective. Statistical adjustments for measured confounding factors were made in eight studies, including three prospective studies. The adjusted confounders differed across studies in number and type. Results from meta-analyses based on adjusted estimates, with or without data from retrospective studies, consistently pointed in the same direction as our earlier findings. There were only small differences in the sizes or the level of statistical significance. Using GRADE, we assessed that our confidence in the effect estimates was very low or low for all outcomes. The adjusted estimates generally show similar obstetric harms from FGM/C as unadjusted estimates do. Thus, the current analyses confirm the findings from our previous systematic review. There are sufficient grounds to conclude that FGM/C, with respect to obstetric circumstances, involves harm.

## 1. Introduction

The World Health Organization (WHO) defines female genital mutilation/cutting (FGM/C) as “all procedures involving partial or total removal of the external female genitalia or other injury to the female genital organs for nonmedical reasons” [1]. While the terminology for this centuries-old practice varies across regions, ideological perspectives, and research frames, the preferred expression by UNICEF and UNFPA is the hybrid term “female genital mutilation/cutting” or FGM/C [2]. The word “mutilation,” while possibly estranging practicing communities, establishes a clear linguistic distinction of FGM/C from male circumcision and signals harm of the practice [1].

According to Wade [3], many of Western efforts to end FGM/C since the term “mutilation” gained growing support in the 1970s have relied on claims that the practice involves physical and mental harm. However, reviews of research findings conducted so far have provided only limited evidence to support this assertion [4–6]. Recently, findings from a large study published in 2006, with women from six African countries, showed that women who had undergone FGM/C were significantly more likely than women without FGM/C to suffer adverse obstetric outcomes [7]. Other recent studies have not confirmed a link between FGM/C and obstetric harm, such as prolonged labor [8] and cesarean section [9].

Given such equivocal assessments and the medical profession's concern particularly with the risk of adverse obstetric events for women who have undergone FGM/C, we recently conducted a systematic review of the evidence for an association between FGM/C and obstetric harm [10]. Our review included 28 comparative studies, that is, studies where the frequency of events in women with FGM/C was compared to the frequency among women with no FGM/C. We found a marked association between FGM/C and the occurrence of a number of obstetric events (prolonged labor, obstetric lacerations, obstetric hemorrhage, and difficult delivery) and concluded that FGM/C significantly increases the risk of delivery complications.

Subsequent to the publication of the technical report [10] and an abridged communication in* Obstetrics and Gynecology International* [11], we were contacted by researchers in the field, who raised two concerns with our analyses, (1) use of unadjusted effect estimates and (2) inclusion of results from retrospective studies. In an attempt to accommodate this criticism, we decided to conduct new analyses taking these concerns into account. Thus, in this paper, we present findings from additional analyses on the association between FGM/C and obstetric events, based on adjusted estimates and/or prospective studies.

## 2. Material and Methods

The steps of the systematic review followed guidelines for systematic reviews, for example, the Cochrane Handbook for Systematic Reviews of Interventions [12] as follows: frame the question for review, identify relevant work, appraise studies' quality, summarize the evidence by use of explicit methodology, and interpret the findings. These methodological steps are detailed in our first report [10], which also includes a description of the 28 comparative studies upon which our meta-analyses were based. For the present analysis, we examined each of those 28 studies for prospective features, that is, whether the women's FGM/C status was assessed before the delivery took place.

Our outcomes of interest were prolonged labor, lacerations, caesarean section, episiotomy, instrumental delivery, hemorrhage, and difficult labor.

Our original systematic review had a broad scope, aiming to assess what is called the population average effect, which, if the estimate were unbiased, would be the effect of the exposure observed in a population with possibly unequal distribution across prognostic characteristics [13]. Thus, in our previously published systematic review, unadjusted estimates were extracted from the included primary studies and combined in meta-analyses when deemed appropriate.

Analyses where adjustments are made for prognostic characteristics provide the exposure effects that would be expected between groups with identical (standardized) combinations of the adjusted covariates [14]. Using adjusted estimates is generally recommended, in order to take differences in prognostic factors between groups into account [15].

Thus, we reanalyzed our findings, using adjusted effect estimates. Specifically, we extracted the adjusted point estimate and the corresponding standard error from all included studies where such estimates were available. Some studies reported more than one adjusted analysis. In those cases we extracted the result from the statistical model that most closely resembled the adjusted models in the other studies. In practice, this meant using the full model, that is, the statistical model that adjusted for most confounders. As in our previous meta-analyses of unadjusted estimates, we aggregated the adjusted results using the generic inverse variance method in RevMan version 5.2. The primary adjusted estimates were almost exclusively reported as odds ratios (ORs) in the included studies. The use of ORs also allowed for comparison of results based on unadjusted and adjusted estimates. ORs greater than 1 indicate increased risk of obstetric complications with FGM/C and ORs less than 1 indicate decreased risk of obstetric complications with FGM/C.

As in the original systematic review, after combining the estimates in meta-analyses, we used GRADE (GRADE-Profiler v3.6) to assess our confidence in the effect estimates [16]. The GRADE system distinguishes between randomized and observational studies. Estimates based on findings from randomized trials are by default graded as “high” level of evidence but can be downgraded. Evidence from observational studies is initially graded as “low” level of evidence and can be either downgraded or upgraded (see [17] and http://gradeworkinggroup.org/). The quality of the evidence is graded high, moderate, low, or very low [17]. The domains used in GRADE for assessing whether to upgrade or downgrade the confidence in estimates of effect are methodological quality of studies, consistency across studies, directness, precision, publication bias, magnitude of association, evidence of a dose-response gradient, and all plausible confounders.

## 3. Results 

### 3.1. Description of Studies

Out of the 28 studies included in our original review [7–9, 18–41], we classified seven as prospective [7, 19, 22, 23, 28, 31, 40]. In these studies, exposure data were gathered from female study participants during an antepartum examination, followed by an assessment of outcome data during the delivery situation. In two additional studies it was unclear whether data were collected prospectively [24, 26]. Adjusted estimates were reported in eight of the 28 studies [7, 8, 22, 27–29, 37, 39]. Four studies reported both unadjusted and adjusted effects [8, 22, 29, 37]. Among the seven prospective studies, three reported adjusted effect estimates [7, 22, 28]. The two studies for which we were uncertain whether to classify as prospective did not report adjusted estimates. In our quality appraisal we judged that the prospective studies ranged from low to high in methodological study quality; that is, there was high to low risk of bias (for a detailed account of all quality assessments, see our main technical report [10]).

### 3.2. Additional Analyses

In Table 1, we present results from analyses based on the following data:

- unadjusted estimates from all studies providing such data, that is, the same as our original analysis (model 1),
- adjusted estimates from all studies providing such data (model 2); this analysis addresses the concern regarding our use of unadjusted estimates,
- adjusted estimates, limited to prospective studies (model 3); this analysis addresses both the concern regarding our use of unadjusted estimates and our inclusion of data from retrospective studies,
- unadjusted estimates from prospective studies reporting adjusted results (model 4); this analysis allows a direct comparison of results based on adjusted estimates (in model 3).

We also conducted analyses based on unadjusted estimates from all prospective studies and on unadjusted estimates from all studies that also provided adjusted estimates. These results were similar to the ones presented below and are available from the first author.

Using GRADE, we assessed that our confidence in the effect estimates was very low for almost all outcomes in most models (Table 2). For the estimates based on adjusted estimates from prospective studies, we assessed our confidence in the estimate as “very low” for one and “low” for three (Table 2).

#### 3.2.1. Prolonged Labor

All our analyses regarding prolonged labor showed that women with FGM/C are at significantly greater risk of experiencing prolonged labor than women with no FGM/C. The details are as follows: the six studies with data on prolonged labor had inconsistent findings. The studies' 12 unadjusted point estimates (ORs) varied between ORs of 0.30 and 3.56. We combined unadjusted estimates from five studies (715,079 women), which resulted in a pooled OR of 1.78 (95% CI = 1.02, 3.11) (model 1). This was a statistically significant result indicating harm from FGM/C, but there was considerable heterogeneity (*I*-squared = 93%).

Four studies reported adjusted ORs for prolonged labor, 13 in total, ranging from 0.20 to 3.40. The number of adjusted confounders differed across studies (from 1 to 12) and type, with maternal age being the only one included in all studies. The pooled estimate of the adjusted estimates gave an OR of 1.49 (95% CI = 1.01, 2.19; *I*-squared = 90%) (model 2).

There was only one prospective study that reported an adjusted estimate for prolonged labor: OR of 2.40 (95% CI = 1.40, 2.80) (model 3) (OR obtained from the author). The unadjusted estimate from the same prospective study was larger: OR of 3.56 (95% CI = 2.85, 4.43).

#### 3.2.2. Tears/Laceration

We detail the results regarding tears/laceration below, but in sum, whereas the pooled result of unadjusted estimates of all studies established a significant difference between the two groups of women, the pooled result of adjusted estimates and from prospective studies failed to establish an equally convincing difference.

There were 14 studies with dichotomous data on obstetric tears/lacerations. The findings were inconsistent, with 39 unadjusted point estimates that varied between 0.15 and 10.2. Combining unadjusted estimates from these 14 studies (738,672 women) resulted in a pooled OR of 1.45 (95% CI = 1.05, 2.00) (model 1). This was a statistically significant result indicating harm from FGM/C, but there was considerable heterogeneity (*I*-squared = 89%).

Four studies reported 18 adjusted ORs for obstetric tears, ranging from 0.75 to 8.80. The adjusted confounders differed across studies in number (2 to 12) and type, with no identical confounders applied across all four studies. However, the adjusted estimates were either stratified by or adjusted for parity such that they reflected obstetric tears among primiparous women. Compared to the unadjusted pooled estimate, the pooled adjusted estimate of four studies resulted in a smaller, nonsignificant OR of 1.39 (95% CI = 0.99, 1.95) and moderate heterogeneity (model 2). No prospective studies presented adjusted estimates for obstetric tears. However, it was possible to aggregate the unadjusted results from five prospective studies. This analysis failed to establish a statistically significant difference between women who had undergone FGM/C and women who had not (OR = 1.69, 95% CI = 0.63, 4.56).

#### 3.2.3. Caesarean Section

Overall, we found that while the pooled result of 15 unadjusted estimates failed to establish a significant difference between women who had and had not been exposed to FGM/C, pooling of adjusted results from two prospective studies suggested a statistically significant difference with respect to cesarean section. The detailed results are as follows: the 15 studies with data on cesarean section had inconsistent findings. There were 57 unadjusted ORs, which varied between 0.52 and 17.6. We combined unadjusted estimates from 15 studies (2.74 million women), which gave a pooled OR of 1.28 (95% CI = 0.95, 1.72) (model 1). This result is based on very heterogeneous data (*I*-squared = 97%) and neither harm nor benefit can be ruled out.

Five studies reported a total of 22 adjusted ORs with point estimates ranging from 0.28 to 3.00, indicating both harm and benefit from FGM/C. The adjusted confounders varied across studies in number (2 to 12) and type. Maternal age was the only confounder that was included across all studies, but the analyses were either stratified by or adjusted for parity. We selected to pool estimates provided for multiparity (we note that results were comparable for primiparous women). The pooled adjusted estimate of all studies that had adjusted estimates resulted in a statistically nonsignificant OR of 1.32 (95% CI = 0.97, 1.80), with considerable heterogeneity (model 2). Eighty-three percent of the variability observed between the studies was attributable to between-study differences and not random variation.

Two prospective studies (about 20,000 women) of variable risk of bias presented adjusted estimates. In pooled analyses they showed a statistically significant result and there was no heterogeneity. The OR was 1.60 (95% CI = 1.33, 1.91) (model 3), indicating a greater risk of cesarean section among women with FGM/C. The unadjusted estimates from these two prospective studies were very different (OR = 4.21 and OR = 0.91). The pooled estimate based on these estimates showed a larger, but nonsignificant, effect and very high heterogeneity (*I*-squared = 100%) (model 4).

#### 3.2.4. Episiotomy

The details are found below, but in summary, the pooling of unadjusted estimates from all possible studies and pooling of unadjusted estimates from prospective studies both indicate a greater risk of episiotomy among women with FGM/C, but the adjusted estimate—from a single study—was less convincing (statistically nonsignificant difference).

Similar to the outcomes reported above, findings from 11 studies were inconsistent for episiotomy. The 28 unadjusted point estimates varied between 0.46 and 2.75. Combining unadjusted estimates from these 11 studies (35,467 women) resulted in a pooled OR of 1.57 (95% CI = 1.00, 2.47) (model 1). This was a (borderline) statistically significant result of harm from FGM/C, and there was very high heterogeneity (*I*-squared = 96%).

Only one (retrospective) study reported adjusted ORs (about 4,000 women). The six reported estimates from this study ranged from 0.73 to 1.18, and none were statistically significant. In each statistical model, stratified by parity and type of FGM/C, an additional covariate was added such that the most inclusive model had 12 covariates. In Table 1, we show the adjusted OR from this study for any delivery among women with FGM/C type II (excision). The result from the most inclusive model showed that neither harm nor benefit from FGM/C could be ruled out (OR 1.18, 95% CI = 0.76, 1.84) (model 2). No prospective studies presented adjusted estimates for episiotomy (model 3). However, it was possible to aggregate the unadjusted results from five prospective studies. The pooled estimate showed a statistically significant OR of 1.70 (95% CI = 1.27, 2.26).

#### 3.2.5. Instrumental Delivery

Overall, with respect to instrumental delivery, the estimates were equivocal. Specifically, there were nine studies with data on instrumental delivery, with inconsistent findings. The 21 point estimates of unadjusted ORs varied between 0.52 and 6.47. Unadjusted estimates from nine studies (2.34 million women) were combined, resulting in a pooled OR of 1.15 (95% CI = 0.77, 1.70) (model 1). This result is based on data from included studies showing considerable heterogeneity (*I*-squared = 91%), and neither harm nor benefit can be ruled out.

Two registry studies (about 705,000 women) reported a total of five adjusted ORs regarding instrumental delivery, with point estimates ranging from 0.9 to 2.1. Both studies adjusted for maternal age. One study also adjusted for parity, while the other stratified primiparous and multiparous women in addition to adjusting for gestational age and birth weight. The pooled adjusted estimate for primiparous women was 1.56 (95% CI = 1.32, 1.86) (model 2). This was a statistically significant result with no heterogeneity. Also the pooled adjusted estimate for multiparous women was in the direction of harm, but benefit could not be ruled out (OR = 1.34, 95% CI = 0.80, 2.26) and there was moderate heterogeneity (*I*-squared = 56%) (model 2).

No prospective studies reported adjusted estimates for instrumental delivery (model 3). However, the pooled unadjusted estimates from four prospective studies showed a nonstatistically significant OR of 1.14 (95% CI = 0.65, 1.99).

#### 3.2.6. Hemorrhage

In general, with the exception of the pooled estimates from prospective studies, the results suggested a greater risk of hemorrhage among women with FGM/C. The detailed results are as follows: there were nine studies with dichotomous data on obstetric or postpartum hemorrhage. The 19 reported point estimates in these studies varied between 0.96 and 13.0. We combined unadjusted estimates from eight studies (746,667 women), which gave a pooled OR of 2.18 (95% CI = 1.40, 3.37) (model 1). This was a statistically significant result indicating a greater risk of postpartum hemorrhage among women with FGM/C, but there was considerable heterogeneity (*I*-squared = 93%).

Five studies reported adjusted odds or risk ratios for hemorrhage, 16 in total ranging from 0.94 to 2.50. There were 2 to 9 adjusted confounders in these studies. No identical confounders were applied across all studies, but three studies adjusted for maternal age. Use of adjusted estimates from five studies gave a pooled estimate of 1.50 (95% CI = 1.22, 1.84) (model 2). Although this estimate showed a weaker association between FGM/C and hemorrhage than the unadjusted pooled estimate, it did show statistically significant harm and less heterogeneity (*I*-squared = 19%).

In contrast to the above result, the pooled adjusted estimate based on the two prospective studies (about 33,000 women) that reported adjusted data for hemorrhage was nonsignificant (OR = 1.91, 95% CI = 0.89, 4.08) and more heterogeneous (*I*-squared = 61%) (model 3). The pooled estimate based on unadjusted estimates from the same two studies was almost identical (OR = 1.98, 95% CI = 0.79, 4.94), but heterogeneity was larger (*I*-squared = 98%).

#### 3.2.7. Difficult Delivery

Our analyses show that regardless of model, all results indicate that women with FGM/C are at significantly greater risk of experiencing difficulties during delivery than women with no FGM/C. The details are as follows: the six studies with dichotomous data on difficult delivery among women with FGM/C and women who had not undergone FGM/C reported five unadjusted point estimates. These ORs varied between 1.20 and 11.5. That is, all were in the direction of harm. Unadjusted estimates from four studies (11,659 women) could be combined. This resulted in a pooled OR of 2.93 (95% CI = 1.30, 6.61), a statistically significant result but with considerable heterogeneity (*I*-square = 92%).

We note that one study compared not having undergone FGM/C with having FGM/C type I, showing adjusted ORs of 0.17 and 0.32 (favoring not having FGM/C). This study had the following covariates: maternal age, number of deliveries, education, religion, marital status, residence, and type of consultation. Further, two studies each reported one adjusted OR (1.22 and 2.30). Common covariates in the two studies were sociodemographic variables, such as age and ethnicity, and one study also included delivery place and birth assistant. The pooled result based on adjusted estimates from the two studies that could be combined resulted in a smaller but significant OR of 1.88 (95% CI = 1.06, 3.35). There was moderate heterogeneity (*I*-square = 49%).

In Table 1, we also show the unadjusted and adjusted ORs from one prospective study that provided data concerning difficult delivery (about 4,800 women). The estimate showed less harm from FGM/C in the adjusted model (OR = 2.30, 95% CI = 1.3, 2.5) than in the unadjusted model (OR = 3.29, 95% CI = 2.37, 4.57), but both estimates were statistically significant.

## 4. Discussion

We aimed to extend the results of our initial systematic review on the obstetric consequences of FGM/C by conducting additional analyses based on adjusted effect estimates from the included studies, particularly prospective studies. In both unadjusted and adjusted aggregated analyses, the results show a strong epidemiological association between female genital mutilation/cutting (FGM/C) and obstetric complications. However, due to the limited quality of the available evidence, we have low confidence that the estimates we report represent the exact size of the effect of FGM/C on the risk of obstetric complications. We did not identify any evidence for benefits from FGM/C.

Conducting the additional analyses using adjusted effect estimates added complexity to the findings. However, adjustment made no difference to the direction and little difference to the size or significance of effects in the pooled analyses. Although the difference was generally small, in all but three instances adjusted analyses reduced the strength of association compared to unadjusted analyses. Moving from unadjusted meta-analyses to adjusted analyses resulted in an average loss of eight studies and about 835,000 participants across the seven outcomes. By limiting our analysis to only prospective studies, we missed data for several outcomes. On the other hand, we observed that heterogeneity consistently decreased as both fewer studies and adjusted estimates were aggregated. Remaining heterogeneity may be due to residual confounding and from other biases that varied across studies.

With respect to prospective studies, which in general can be assumed to provide stronger evidence of effects [42], only three of the seven studies with prospective features presented adjusted effect estimates. The studies had variable risks of bias; only results from two studies could be pooled in meta-analysis, and for the two outcomes which could be aggregated, the adjusted confounders differed in both numbers and types. Instead of combining these two studies in a meta-analysis, we could have relied on the one prospective study that we considered to have a low risk of bias [7]. This would not have had much influence on our findings: the estimated association between FGM/C and caesarean section would remain significant but would be smaller, and for hemorrhage the estimate would also be smaller but would become statistically significant.

The process of conducting additional analyses using adjusted effect estimates was complex. While most studies reported no adjusted estimates, others reported multiple adjusted estimates from analyses including different sets of covariates. Overall, there was great variation with respect to the measurement, inclusion, methods of analysis, and reporting of confounders. Accounting for these variations in our systematic review was challenging and time-consuming; whether to aggregate estimates at all was extensively debated. In the end, our current findings are not more conclusive than those from our previous analysis and generally show largely similar degrees of obstetric harm from FGM/C. Thus, we still find it reasonable to conclude that there is convincing evidence that FGM/C is associated with an increased risk of obstetric complications. However, the available evidence does not allow for firm conclusions about how strong this relationship is.

Observational studies are inherently limited by confounding which is unlikely to be fully adjusted for. However, observational studies may still provide convincing evidence of causal relationships, for example, when all important confounding factors can be taken into account and adjusted for (researchers can only adjust for known confounders) [15]. Of those eight studies that did report adjusted estimates, maternal age and parity were commonly considered as confounders, but choice of included confounders was highly variable across studies. Thus, there is clearly no consensus among FGM/C researchers as to which factors constitute important confounders when estimating the association between FGM/C and obstetric events. The effect of unknown confounders may be operating in either direction, within and across all of the included studies [43].

## 5. Conclusion

This analysis has presented a comprehensive set of meta-analyses on the obstetric consequences of FGM/C, taking adjusted effect estimates and prospective features of studies into account. As in our original systematic review and meta-analysis, we found that there is uncertainty about the size of the greater obstetric risk of harm among women with FGM/C relative to women with no FGM/C but sufficient grounds to conclude that FGM/C involves obstetric harm.

## Figures and Tables

**Table 1:** Meta-analyses results.

Outcomes	Model 1 Unadjusted results. All studies	Model 2 Adjusted results. All studies reporting adjusted results	Model 3 Adjusted results. Prospective studies reporting adjusted results	Model 4 Unadjusted results. Prospective studies reporting adjusted results
Prolonged labor	OR = 1.78 (1.02, 3.11) *I* ^2^ = 93% (*k* = 5, *n* = 715079)	OR = 1.49 (1.01, 2.19) *I* ^2^ = 90% (*k* = 4, *n*-max = 715333)	OR = 2.40 (1.40, 2.8) (*k* = 1, *n*-max = 4800)	OR = 3.56 (2.85, 4.43) *I* ^2^ = NA (*k* = 1, *n* = 4800)

Tears/lacerations	OR = 1.45 (1.05, 2.00) *I* ^2^ = 89% (*k* = 14, *n* = 738672)	OR = 1.39 (0.99, 1.95) *I* ^2^ = 55% (*k* = 4, *n*-max = 714502)	*k* = 0	*k* = 0

Caesarean section (multi)	OR = 1.28 (0.95, 1.72) *I* ^2^ = 97% (*k* = 15, *n* = 2742305)	OR = 1.32 (0.97, 1.80) *I* ^2^ = 83% (*k* = 5, *n*-max = 743435)	OR = 1.60 (1.33, 1.91) *I* ^2^ = 0% (*k* = 2, *n*-max = 20354)	OR = 1.85 (0.37, 9.27) *I* ^2^ = 100% (*k* = 2, *n* = 20354)

Episiotomy	OR = 1.57 (1.00, 2.47) *I* ^2^ = 96% (*k* = 11, *n* = 35467)	OR = 1.18 (0.76, 1.84) *I* ^2^ = NA (*k* = 1, *n*-max = 4054)	*k* = 0	*k* = 0

Instrumental delivery	OR = 1.15 (0.77, 1.70) *I* ^2^ = 91% (*k* = 9, *n* = 2343966)	OR primi = 1.56 (1.32, 1.86) *I* ^2^ = 0% (*k* = 2, *n*-max = 705128) OR multi = 1.34 (0.80, 2.26) *I* ^2^ = 56% (*k* = 2, *n*-max = 705672)	*k* = 0	*k* = 0

Hemorrhage	OR = 2.18 (1.40, 3.37) *I* ^2^ = 93% (*k* = 9, *n* = 746667)	OR = 1.50 (1.22, 1.84) *I* ^2^ = 19% (*k* = 5, *n*-max = 743641)	OR = 1.91 (0.89, 4.08) *I* ^2^ = 61% (*k* = 2, *n*-max = 33193)	OR = 1.98 (0.79, 4.94) *I* ^2^ = 98% (*k* = 2, *n* = 33193)

Difficult labor	OR = 2.93 (1.30, 6.61) *I* ^2^ = 92% (*k* = 4, *n* = 11659)	OR = 1.88 (1.06, 3.35) *I* ^2^ = 49% (*k* = 2, *n*-max = 5907)	OR = 2.30 (1.3, 2.5) (*k* = 1, *n*-max = 4800)	OR = 3.29 (2.37, 4.57) *I* ^2^ = NA (*k* = 1, *n* = 4800)

OR: odds ratio with 95% confidence interval. For caesarean section and postpartum blood loss one study [7] provided adjusted RR not OR, which may have slightly lowered the pooled point estimate; *k*: number of studies; *n*: number of participants; *n*-max: maximum possible number of study participants included in analysis.

**Table 2:** Summary of GRADE assessments.

Outcomes	Model 1: unadjusted results. All studies	Model 2: adjusted results. All studies reporting adjusted results	Model 3: adjusted results. Prospective studies	Model 4: unadjusted results. Prospective studies
Prolonged labor	⊕⊝⊝ ⊝ **very low** ^1,2^	⊕⊝⊝ ⊝ **very low** ^9,2^	⊕⊕⊝ ⊝ **low** ^15^	⊕⊝⊝ ⊝ **very low** ^16,2^
Tears/lacerations	⊕⊝⊝ ⊝ **very low** ^3,2^	⊕⊝⊝ ⊝ **very low** ^9^	—	—
Caesarean section	⊕⊝⊝ ⊝ **very low** ^4,2,5^	⊕⊝⊝ ⊝ **very low** ^10,2^	⊕⊕⊝ ⊝ **low**	⊕⊝⊝ ⊝ **very low** ^2,5^
Episiotomy	⊕⊝⊝ ⊝ **very low** ^6,2^	⊕⊝⊝ ⊝ **very low** ^11,12^	—	—
Instrumental delivery	⊕⊝⊝ ⊝ **very low** ^7,2,5^	⊕⊝⊝ ⊝ **very low** ^13^	—	—
Hemorrhage	⊕⊝⊝ ⊝ **very low** ^8,2^	⊕⊝⊝ ⊝ **very low** ^14^	⊕⊝⊝ ⊝ **very low** ^2,5^	⊕⊝⊝ ⊝ **very low** ^2,5^
Difficult labor	⊕⊝⊝ ⊝ **very low** ^9,2,5^	⊕⊕⊝ ⊝ **low**	⊕⊕⊝ ⊝ **low** ^15^	⊕⊝⊝ ⊝ **very low** ^16^

**GRADE working group grades of evidence:  High quality:** further research is very unlikely to change our confidence in the estimate of effect. **Moderate quality:** further research is likely to have an important impact on our confidence in the estimate of effect and may change the estimate. **Low quality:** further research is very likely to have an important impact on our confidence in the estimate of effect and is likely to change the estimate. **Very low quality:** we are very uncertain about the estimate.

^1^5 of 5 studies had low methodological study quality (one additional study includes this outcome, but we have not received the data).

^2^Considerable heterogeneity indicated by *I* ^2^ showed inconsistency across studies.

^3^12 of 14 studies had low methodological study quality (one additional study includes this outcome, but we have not received the data).

^4^12 of 15 studies had low methodological study quality.

^5^CI is wide and crosses limitations of precision.

^6^9 of 11 studies had low methodological study quality.

^7^7 of 8 studies had low methodological study quality.

^8^6 of 8 studies had low methodological study quality (one additional study includes this outcome but we have not received the data).

^9^3 of 4 studies had low methodological study quality.

^10^4 of 5 studies had low methodological study quality.

^11^The study had low methodological study quality.

^12^Single study.

^13^2 of 2 studies had low methodological study quality.

^14^3 of 5 studies had low methodological study quality.

^15^Only one study of low to moderate methodological study quality but fairly large sample size and large effect estimate.

^16^Only one study of low to moderate methodological study quality, unadjusted results, but fairly large sample size and large effect estimate.
